# Foamed Geopolymer Composites with the Addition of Glass Wool Waste

**DOI:** 10.3390/ma14174978

**Published:** 2021-08-31

**Authors:** Barbara Kozub, Patrycja Bazan, Rihards Gailitis, Kinga Korniejenko, Dariusz Mierzwiński

**Affiliations:** 1 Department of Materials Engineering, Faculty of Material Engineering and Physics, Cracow University of Technology, Jana Pawła II 37, 31-864 Cracow, Poland; barbara.kozub@pk.edu.pl (B.K.); patrycja.bazan@gmail.com (P.B.); dariusz.mierzwinski@pk.edu.pl (D.M.); 2 Faculty of Civil Engineering, Riga Technical University, Kipsalas 6A/B, LV-1048 Riga, Latvia; rihards.gailitis@edu.rtu.lv

**Keywords:** foamed geopolymer, glass wool waste, fly ash, thermal conductivity coefficient, thermal radiation changes

## Abstract

This study examines foamed geopolymer composites based on fly ash from the Skawina coal-fired power plant in Poland. The paper presents the effect of adding 3% and 5% by weight of glass wool waste on selected properties of foamed geopolymers. The scope of the tests carried out included density measurements, compressive and bending strength tests, measurements of the heat conduction coefficient, and the results of measurements of changes in thermal radiation in samples subjected to a temperature of 800 °C. The obtained results indicate that glass wool waste can be successfully used to lower the density and heat conduction coefficient of foamed geopolymer composites with a fly ash matrix. In addition, the results of changes in thermal radiation in the samples subjected to the temperature of 800 °C showed a positive effect of the addition of glass wool waste. Moreover, the introduction of the addition of glass wool waste made it possible to increase the compressive strength of the examined foamed geopolymers. For the material modified with 3% by weight of mineral wool, the increase in compressive strength was about 10%, and the increase in fibers in the amount of 5% by weight resulted in an increase of 20% concerning the base material. The obtained results seem promising for future applications. Such materials can be used in technical constructions as thermal insulation materials.

## 1. Introduction

One of the goals of sustainable development policy is designing new energy-efficient buildings, including the selection of appropriate materials for thermal insulation, ensuring savings of energy used for heating and cooling buildings and having a minimal negative impact on the environment. Glass wool (GW) and stone wool (SW) are among the most popular materials in the world used for thermal insulation [1,2,3,4]. Unfortunately, the production process of mineral wool is not a waste-free process. As a result, by-products are created, such as cuttings of mineral wool. In 2010, the estimated amount of waste generated during the production of mineral wool in 27 European Union countries amounted to 2.3 million tonnes [4,5]. Waste from insulation materials, e.g., from construction sites or demolition of buildings, is considered to be practically impossible to recycle [6]. Nevertheless, some studies indicate the possibility of using mineral wool waste in cement [4,7,8] and cement composites [9,10,11], gypsum boards [4,9,10], or fiber composites [4]. Cheng et al. investigated the effect of the addition of mineral wool on the properties of cement composites. The test results showed that partial replacement of cement with mineral wool waste improves physical, mechanical, and abrasive properties. These properties are due to the higher density of the material due to the filling effect of the pozzolana product. The results presented suggest that mineral wool waste may act as a cementitious material or inert filler in cement-based composites. The addition of 10% by volume of mineral wool increased the compressive strength by about 20% and the tensile strength by about 30%. In addition, scientists pointed to differences in the effects due to particle size. The larger particles act as an anti-crack propagation agent, while the smaller particles can cement due to hydration or the pozzolanic reaction [9].

The literature [12,13,14,15] also includes studies showing the possibility of using waste stone and glass wool as a binder for geopolymers. Murri et al. studied metakaolin-based geopolymer composites reinforced with glass wool. The mechanical and thermal insulation properties of the produced composites were characterized. The wool content was 23% and 31% by volume. The composites showed an average density of 1.0 g/cm^3^, a thermal conductivity of 0.2 W/mK, and compressive and flexural strength of about 9 and 5 MPa, respectively. The strength properties and fracture toughness increased after the addition of mineral fibers. The results showed that composites with mineral wool with 23% vol. wool fibers are suitable as a fireproof barrier [16].

Many binders are used in the production of mineral wool, including sodium silicates, polyesters, melamine urea with formaldehyde, polyamides, furan-based resins, and others, but phenolic resin binders are indicated as the preferred binder for mineral wool. Glass wool, also known as fiberglass or fiberglass wool, is made from sand, limestone, soda, and borax. In recent years, the production process has used more and more amounts of recycled glass, up to 90%, and the rest are the additives: sand, limestone, soda, and borax. To achieve the required properties of mineral wool, the amount of binder and glue is variable. The amount of binder gives the mineral wool its strength properties [17].

Mineral wool waste is characterized by a large specific surface, and in X-ray radiation, it is amorphous, which increases the reactivity of wool in an alkaline environment [5,18,19].

Geopolymers are inorganic amorphous synthetic aluminosilicate polymers. They are formed as a result of the synthesis of silicon and aluminum as well as geologically obtained minerals. In the spatial structure of geopolymeric materials, there appear SiO_4_ and AlO_4_ tetrahedrons characteristic for this type of materials [20,21,22,23].

Geopolymeric materials are becoming more common due to the production of a carbon dioxide-reduced binder and other products using waste materials such as mineral wool. The conducted research shows that mineral wool geopolymers generate about 80% less CO_2_ emissions compared to ordinary concrete. Such materials can be used for the production of fiber-reinforced panels, acoustic panels, paving slabs, and facade elements. In recent years, the fact that Europe’s mineral deposits are largely depleted has been strongly emphasized, and economic growth is generating huge amounts of construction and demolition waste, which is an important environmental problem. It is estimated that in Europe, demolition waste accounts for about 30% of all waste, and multi-material materials can be recycled to some extent. The EC Waste Directive says in Art 11-2b that by 2020, the preparation of materials for reuse, recycling, and other recovery and the use of waste to replace other materials will be increased to 70% by weight [24].

In recent years, based on foam concrete, attention has been focused on the development of foams with geopolymers. These materials are characterized by low density and thus, relatively high strength and thermal stability. Due to these properties, closed-cell foam materials have application potential in the field of construction and as fire-resistant materials. Open-pore foams, on the other hand, can be used as filtering materials [25,26,27].

Geopolymer foams are materials that result from mixing a chemical blowing agent with a geopolymeric mass. Hydrogen peroxide is most often used as a blowing agent, but the greater the porosity of geopolymer foams, the lower the material’s strength [28].

The study investigated foamed geopolymer composites with the addition of waste glass wool. The main objective of the research was to investigate whether the use of the addition of glass wool waste affects the strength and thermal properties of the produced materials, which determines the possibility of using the produced geopolymer composites for engineering structures. In addition, nowadays, the requirements for new materials are extremely high, in terms of strength and thermal and ecological properties. The use of waste materials is a step towards taking care of the natural environment. Composite geopolymer foams that meet the above requirements may soon become a widely used material.

## 2. Materials and Methods

### 2.1. Materials and Sample Preparation

The geopolymer matrix consisted of fly ash from the Skawina Combined Heat and Power Plant (Skawina, Poland) and fine-grained sand with a saturated surface (the surfaces of the sand grains are “dry”, but the cavities between the particles are saturated with water—no surface absorption) in a one-to-one ratio.

**Figure 1:**
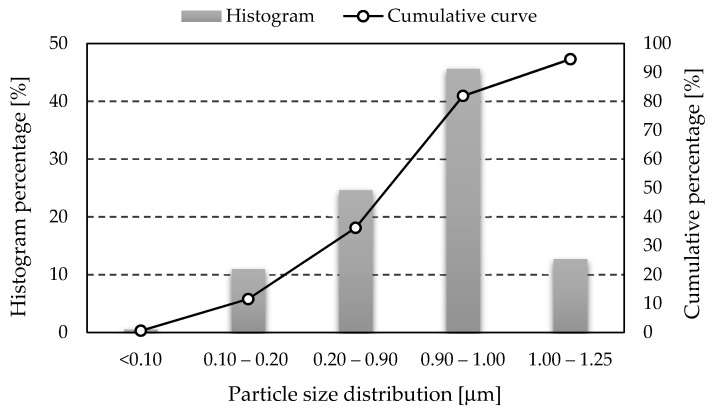
Histogram of particle size distribution and cumulative particle size distribution curve for the fly ash used in investigation.

**Figure 2:**
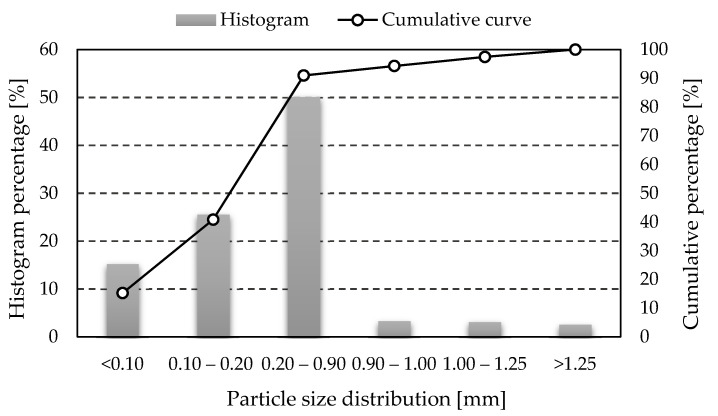
Histogram of particle size distribution and cumulative particle size distribution curve for the construction sand used in the investigation.

Based on the ASTM C618-19 standard [29], depending on the percentage of chemical and mineralogical composition, two ash classes are distinguished: F and C. In this work, class F ash was used, for which the chemical composition requirements are presented in Table 1 [30]. It consists mainly of aluminum oxide (Al_2_O_3_) and silicon dioxide (SiO_2_) and contains less than 4% calcium oxide (CaO). The exact percentage of the individual phases in the used fly ash has been determined in previous studies [31]. This type of fly ash is characterized by certain physical and chemical properties that support the geopolymerization process [32,33]. Figure 1 and Figure 2 show the histograms of the particle size distribution and the cumulative particle size distribution curves used in the investigation fly ash and construction sand (results obtained from own research).

**Figure 3:**
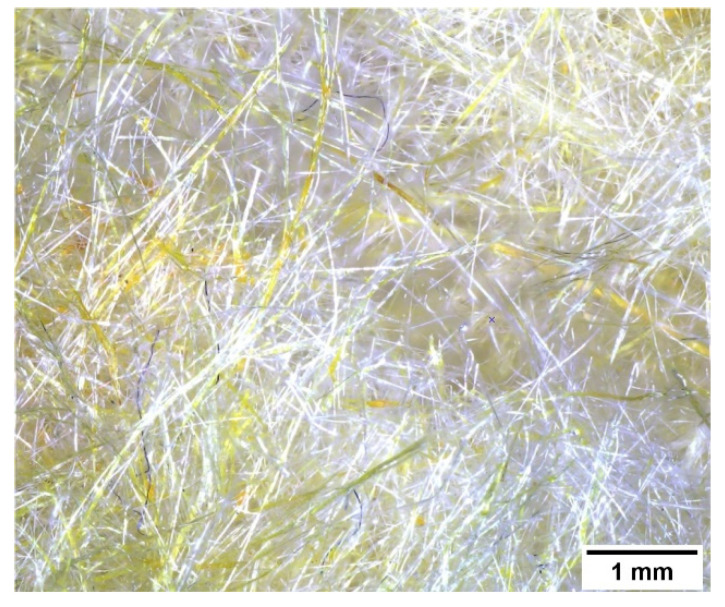
Glass wool waste added to geopolymer composites.

Glass wool waste was obtained from the demolition of the renovated campus building at the Cracow University of Technology (Cracow, Poland). Waste glass wool samples were separated manually from the C&D waste piles. The exact date of the installation of this old glass wool is not known exactly, but it is estimated that it could have been in the 1990s. Figure 3 shows a microscopic photo of the waste glass wool used for testing.

Glass wool waste was added to geopolymer composites in the amount of 3% and 5% by weight. For comparison purposes, a reference sample based on matrix material without any additives (samples marked as 0% GW) was made. As an alkaline activator, 10-molar sodium hydroxide solution and sodium water glass R-145 (2.5 molar modulus; density about 1.45 g/cm^3^), combined in a ratio of one to two, were used. As a blowing agent in the production of foamed geopolymers, two chemical foaming agents (3% by weight of hydrogen peroxide and 0.5% by weight of aluminum powder) were added to a fly ash-based geopolymer matrix. Table 2 presents a list of the produced composites. The volumetric share was calculated based on formula (1) [34]:

(1) $$VF_{n}=\frac{1}{1+\frac{\rho_{f}}{\rho_{m}}\left(\frac{1}{wt}-1\right)}$$

where:

$\rho_{f}$—filler density;

$\rho_{m}$—matrix density;

wt—weight fraction of filler;

glass wool density is 0.48 g/cm^3^ [35].

To prepare an alkaline solution, an aqueous solution of sodium silicate and water was added to the solid sodium hydroxide (tap water was used instead of distilled water). The solution was then mixed thoroughly and allowed to equilibrate its temperature with the ambient temperature (which took about 2 h). In the next step, to prepare geopolymer masses, fly ash, construction sand, glass wool waste, and alkaline solution were mixed for about 15 min, then the foaming agents were added and further mixed for about 5 min until a homogeneous paste was obtained. In the final step, the prepared masses were transferred to the molds. Molded geopolymer composites were heated in a laboratory dryer (SLW 750 STD, POL-EKO-APARATURA, Wodzisław Śląski, Poland) for 24 h at 75 °C in the atmospheric pressure. The prepared samples were tested after 28 days.

### 2.2. Density

The density of the samples was determined by a geometric method before performing the strength test. Density was determined as the arithmetic means of the measurements for six cuboidal samples for compressive strength tests for each of the analyzed compositions of the studied geopolymer composites. For the obtained results, the standard deviation was determined (marked in the graphs as error bars). The dimensions of each sample were measured with a laboratory caliper (measuring accuracy of 0.01 mm) and the weight of the samples was measured on the RADWAG PS 200/2000.R2 laboratory precise analytical balance (maximum load: 200/2000 g; reading accuracy: 0.001/0.01 g) (Radwag Wagi Elektroniczne, Radom, Poland).

### 2.3. Strength Tests

Since there is no separate standard for geopolymer materials, the compressive strength test was carried out according to the method described in the concrete standard EN 12390-3 (“Testing hardened concrete. Compressive strength of test specimens”) [36]. The tests were carried out on a universal strength testing machine Matest 3000 kN at a rate of 0.05 MPa/s. Six cuboidal samples of dimensions (approximately) 50 mm × 50 mm × 50 mm were prepared and tested for all analyzed chemical compositions of the geopolymer composite. For the obtained results, the standard deviation was determined (marked in the graphs as error bars).

As with the compressive strength test, for flexural strength tests the concrete standard EN 12390-5 (“Testing hardened concrete. Flexural strength of test specimens”) [37] was also used. The flexural strength tests were also carried out on a universal testing machine Matest 3000 kN at a rate of 0.05 MPa/s. Four prism specimens with dimensions (approximately) 200 mm × 50 mm × 50 mm with a support distance of 150 mm were prepared and tested for all analyzed geopolymer composites.

### 2.4. Porosity

The porosity measurements were carried out based on photos of the samples and processed with ImageJ using the Fast Optical Porosity Measurement (TOP) method. The method uses a macro file (jPOR.txt) for ImageJ [38]. The method does not require any special scientific equipment and can be fully run using free software. The digital images are obtained with a conventional scanner, although the technique can be used for any high-resolution digital image obtained by various means (e.g., flat scan, digital capture).

### 2.5. Thermal Conductivity

To determine the thermal conductivity coefficient λ, measurements were carried out using the HFM 446 Lambda Series device from NETZSCH (Netzsch GmbH & Co., Selb, Germany), following the ASTM C518 JIS A1412 [39], ISO 8301 [40], and DIN EN 12667 [41] standards. Samples with dimensions 200 mm × 200 mm × 25 mm were prepared for the tests. Four repetitions of measurements were made for each of the studied geopolymers. The measurement consisted of placing the sample between two heated plates set to different temperatures. The heat flow through the sample was measured using a calibrated heat flux converter. The measurement was performed after achieving thermal equilibrium. Only the center of the sample measuring 100 mm × 100 mm was used for the analysis.

### 2.6. Thermal Radiation Measurement

The research of thermal radiation measurement was carried out according to the original idea of the employees of the Department of Materials Science at the Cracow University of Technology presented in the previous research in which the results of thermal radiation measurement of geopolymer composites based on fly ash with the addition of melamine fibers were presented [42].

**Figure 4:**
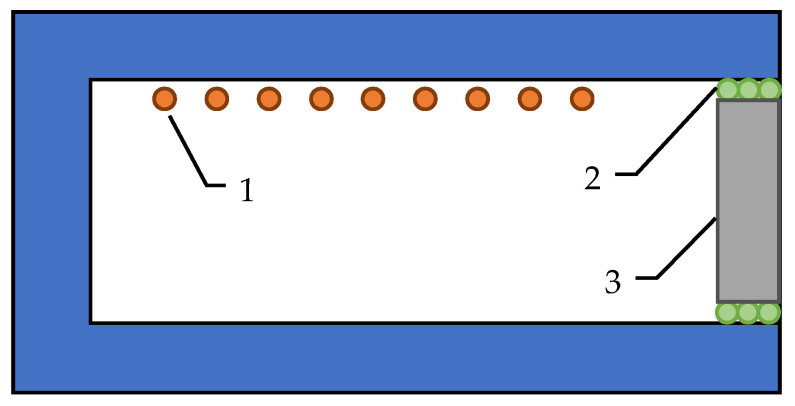
Scheme of the measuring system: (1) heating element, (2) high-temperature insulation material, (3) geopolymer sample in the form of plates with dimensions of 100 mm × 150 mm × 50 mm [33].

The samples in the form of plates with dimensions of 100 mm × 150 mm × 50 mm were placed in a silite electric chamber furnace. The tiles were an insulating element, as shown in the diagram below (Figure 4). As a sealing element, filling the space between the tested plate and the walls of the furnace chamber, an element made of insulating material was used that can withstand temperatures up to about 1500 °C.

**Figure 5:**
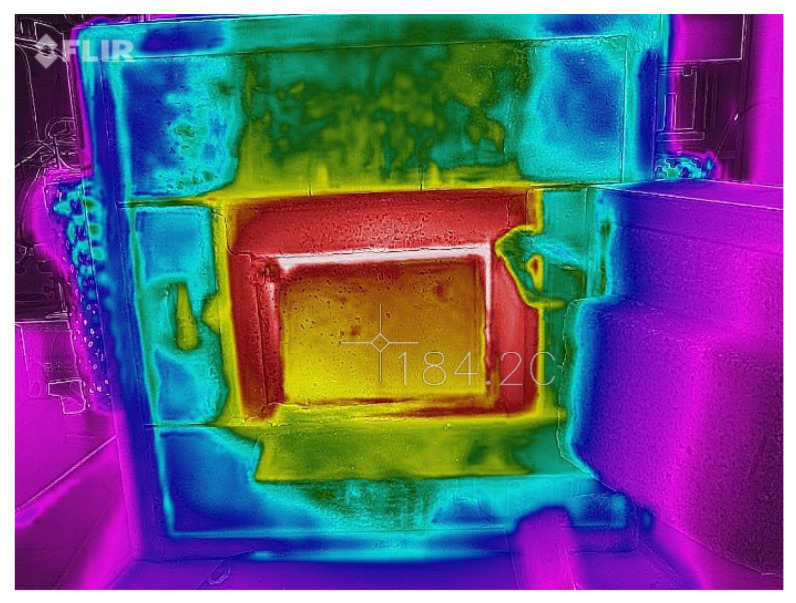
Sample photo of the measurement of thermal radiation.

Changes in thermal radiation were examined on the outer surface of the sample (Figure 5) with the use of a FLIR thermal imaging camera with a field of view (FOV) ≥ 38°, thermal sensitivity < 70 mK, measured infrared wavelength range in the range of 7–14 µm and pixel size < 15 µm. The camera was set at a distance of 1.5 m from the furnace in which the sample was placed. Measurements were made in the center of the sample at a frequency of 60 s for the first hour, with the next reduction in the frequency of the measurement readings until temperature stabilization was achieved. The measurement was completed after three hours.

## 3. Results

### 3.1. Density

**Figure 6:**
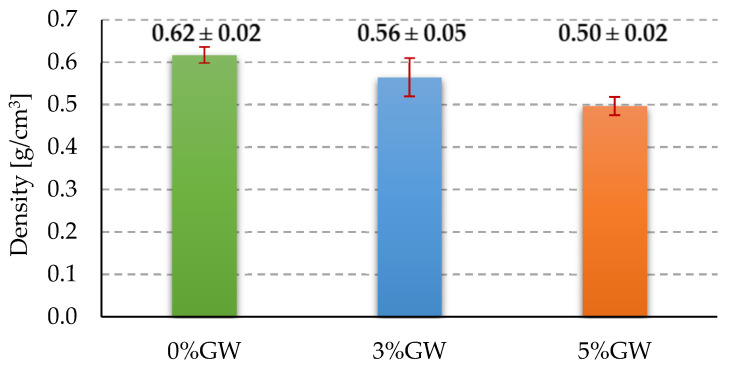
Density results of tested materials.

Figure 6 presents the obtained results of density measurements for all tested foamed geopolymer composites. The mean density value of the fly ash-based foamed geopolymer without any additives was approximately 0.62 g/cm^3^. The addition of glass wool waste made it possible to reduce the density of the foamed geopolymer based on fly ash. The density of foamed geopolymers decreased with an increase in the proportion of the added additive and it amounted to approximately 0.56 g/cm^3^ (−9.7% concerning plain geopolymer) and 0.49 g/cm^3^ (−20% concerning plain geopolymer) for 3% and 5% by weight addition of glass wool waste, respectively.

### 3.2. Strength Tests

**Figure 7:**
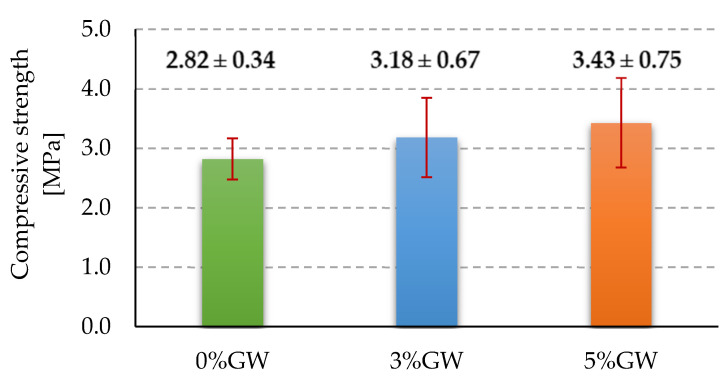
Compressive strength of tested materials.

Figure 7 shows the results of the compressive strength measurements for all tested foamed geopolymer composites. The mean value of the compressive strength for the reference sample was approximately 2.82 MPa. The introduction of the glass wool waste addition made it possible to increase the compressive strength of the tested foamed geopolymers by about 12.8% and 21.6% in relation to the reference sample for 3% and 5% by weight addition of glass wool waste, respectively. The greater the amount of waste glass wool added, the higher the value of the compressive strength was obtained. For the foamed composite with the addition of 3% by weight of glass wool waste, the value of the compressive strength was about 3.18 MPa, while for the amount of 5% by weight of addition, the value of the compressive strength was about 3.43 MPa.

In the case of measurements of flexural strength, all of the tested samples had a flexural strength of less than 1 MPa, which made it impossible to record the measurements and present the results, which is common for foamed geopolymers.

### 3.3. Porosity

**Figure 8:**
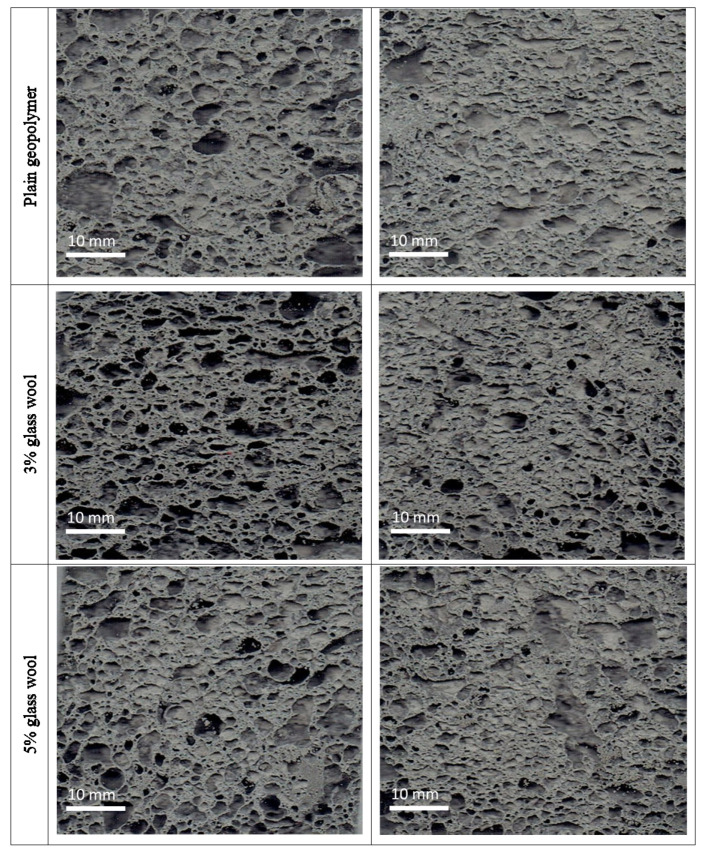
The porous structure of the produced geopolymers.

Figure 8 presents photos of the porous structure of the produced geopolymers, and Table 3 presents the average porosity results from nine photos for each series.

Analyzing the obtained results, the porosity of the produced materials was about 50% and the unmodified material without reinforcing fibers was characterized by the lowest porosity, while with the increase in the proportion of fibers, the porosity of the geopolymeric composite slightly increases, but attention should be paid to the standard deviation, which, in the case of unmodified geopolymer and composite with 3% by weight of the fibers, is relatively high. However, it can be concluded that the introduction of reinforcing fibers does not damage the foam structure. The maximum pore size was about 10 mm, but for the most part, the pore size was in the range of 100–300 µm. The pore size depends on many factors such as the foaming agents used or stabilizers.

### 3.4. Thermal Conductivity

**Figure 9:**
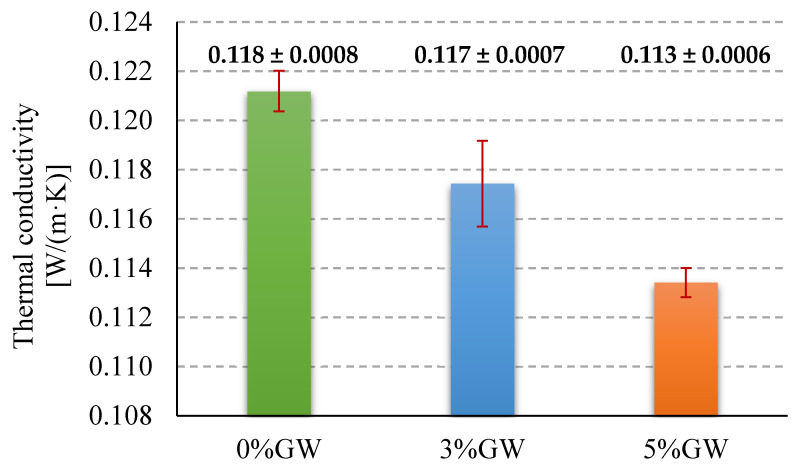
Thermal conductivity coefficient of tested materials.

Figure 9 shows the values of the thermal conductivity coefficient for all tested geopolymers. The highest value of the thermal conductivity coefficient was obtained for pure geopolymer, and it amounted to about 0.121 W/(m·K). The use of the addition of glass wool waste reduced the value of the thermal conductivity coefficient and the amount of 5% by weight of glass wool waste addition to geopolymer matrix reduced the value of the thermal conductivity coefficient to the level of about 0.113 W/(m·K), which was about −6.6% in relation to the reference sample.

### 3.5. Thermal Radiation Measurement

**Figure 10:**
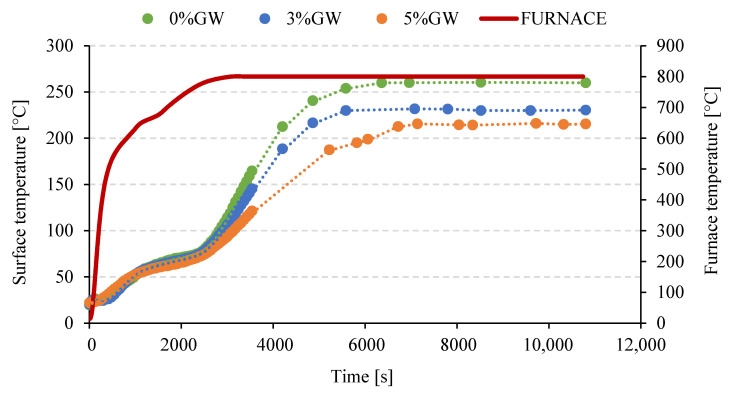
Temperature change depending on the heating time and the glass wool waste content.

Figure 10 shows a graph of changes in temperature measured on the outer surface of foamed geopolymer plates with different content of glass wool waste as a function of time. The green curve corresponds to the reference sample, while the blue and orange curves correspond to the composite with 3% and 5% by weight of glass wool waste, based on the total weight of the geopolymer matrix, respectively. By analyzing the obtained curves, significant differences in the changes in the emission of thermal radiation with the increase in temperature in the furnace can be noticed. Compared to pure foamed geopolymer, both curves for geopolymer composites are characterized by a lower temperature on average by about 30 °C and 45 °C for 3% and 5% by weight addition of glass wool waste, respectively.

**Figure 11:**
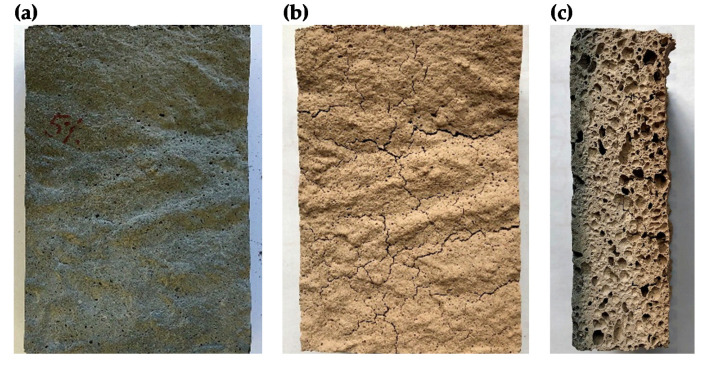
Exemplary photos of the surface of a sample with the addition of 5 wt.% waste glass wool before 8 (**a**) and after 8 (**b**,**c**) exposure to a temperature of 800 °C.

Figure 11 shows example photos of the surface of a sample with the addition of 5 wt.% waste glass wool before and after exposure to a temperature of 800 °C. Discoloration and numerous surface cracks could be observed on the surface of the samples.

## 4. Discussion

This paper presents foamed geopolymer composites with a matrix based on fly ash with the addition of 3% and 5% by weight of glass wool waste. The results obtained for the density measurements were in line with the expectations, namely, the addition of glass waste resulted in a decrease in the density of the composite—this decrease was the higher the greater was the mass fraction of the additive in the composite. The decrease in density after the addition of mineral wool to geopolymers was also noted by other scientists in similar research [12,13,14].

The results of the porosity measuring of the produced materials show that with the increase in the proportion of fibers, the porosity of the geopolymeric composite slightly increases and the introduction of reinforcing fibers does not damage the foam structure. Ji et al. investigated the effect of porosity on the mechanical properties of geopolymers, and the effect of various surfactants and stabilizers on the porosity of foamed geopolymers. Studies have shown that hydrogen peroxide used as a blowing agent for the preparation of foam structures can be successfully used to control and adjust cellular structures in geopolymers. Hydrogen peroxide causes pore growth and also increases the number of macropores (>500 µm). As the content of H_2_O_2_ increases, gas production increases, which leads to the formation of large and open-cell structures [43].

The size and distribution of cellular structures have a significant influence on the mechanical properties of foamed materials. In foamed geopolymers without a stabilizing agent, the blisters are unstable and collapse easily, which in turn leads to a reduction in strength properties. When stabilizers are introduced, the particles absorbed at the interface reduce the surface tension, which in turn stabilizes the foam bubbles [44,45].

The compressive strength test results indicate that the introduction of the glass wool waste addition allows for increasing the compressive strength of the tested foamed geopolymers. As in the case of the results obtained for the density measurements, also in the case of the compressive strength, a certain dependence can be observed that the greater the addition of glass wool waste, the higher the value of the compressive strength recorded during the tests. The best result was achieved for a 5% by weight amount of the addition of glass wool waste, approximately 3.43 MPa, compared to 2.82 MPa for a reference sample without any addition. Erofeev et al. [15], in their work, presented data where for lightweight geopolymers made of mineral wool production wastes, the compressive strength of the samples was in the range of 2.8 to 5.4 MPa. The compressive strength values obtained in this study are similar to those presented in the literature [13,46,47,48,49,50,51] for samples based on slags of ferrous and non-ferrous metallurgy, ashes, and glass wastes.

The results obtained from the measurements of thermal conductivity were in line with the expectations, namely the thermal conductivity of geopolymer composites decreased with the increase in the content of glass wool wastes addition in the tested samples. The use of the 5% by weight of glass wool waste addition reduced the value of the thermal conductivity coefficient for tested geopolymer composite by about 6.6% (from 0.121 to 0.113 W/(m·K)) compared to reference sample without any addition. The obtained values of the thermal conductivity are lower than those reported in the literature [14] for lightweight geopolymers made of mineral wool production wastes, which were in the range from 0.208 to 0.332 W/(m∙K). However, the samples tested in the aforementioned article were characterized by a higher degree of foaming and, as a result, much lower density values (the material density from 800 to 1100 kg/m^3^), which most likely resulted in higher values of the thermal conductivity coefficient.

The thermal radiation measurements tests depending on the used amount of the glass wool waste addition show significant differences in the changes in the emission of thermal radiation with the increase in temperature in the furnace for tested samples. The use of a 5% by weight addition of glass wool waste reduced the registered temperature on the outer surface of the sample by 45 °C in comparison with the temperature registered on the outer surface of the sample made of pure geopolymer. These obtained results seem to be promising for possible applications of tested geopolymer composites for thermal insulations.

## 5. Conclusions

The paper presents foamed geopolymer composites with the addition of glass wool waste. The obtained results indicate that glass wool waste can be successfully used as a reinforcement of geopolymer foams because it has a positive effect on the strength properties by increasing the compressive strength, which, in the case of foamed materials, is an extremely important property. The addition of fibers reduces the density of materials as well as improves thermal properties. Thermal radiation studies in samples subjected to the temperature of 800 °C showed that the addition of glass wool fibers reduces the thermal effect on the material concerning the unmodified material. These results seem promising for possible applications of foamed geopolymer composites with added glass wool waste for thermal insulation applications. In addition, the growing concern about environmental pollution causes a search for new methods of recycling materials, which, until now, were only non-recyclable waste. The presented research results suggest the possibility of using waste such as mineral wool in the production of geopolymers, not only in recycling methods as an eco-friendly material, but also to increase the desired strength properties. However, practical applications require further detailed research to optimize the mechanical properties of foamed geopolymer composites, such as water absorption or resistance to water in various environments and at variable temperatures, which will be the next stage of research.

## Figures and Tables

**Table 1:** Requirements for the chemical composition of class F ash [30].

Type of Fly Ash	Content of Basic Ingredients (%)			
	SiO_2_ + Al_2_O_3_ + Fe_2_O_3_	SO_3_	Loss of Irrigation	Moisture
F class	min. 70	max. 5	max. 6	max. 3

**Table 2:** Determination of samples, weight and volume fractions of fillers.

Designation of Sample	The Proportion of Solid Components (% by Weight/by Volume)		
	Fly Ash	Sand	Glass Wool Waste
0% GW	50	50	0
3% GW	48.5	48.5	3/3.82
5% GW	47.5	47.5	5/6.34

**Table 3:** The average porosity results of the produced geopolymers.

Type of Tested Sample	Average Porosity (%)
Plain Geopolymer	45.3 ± 6.1
3% glass wool	48.0 ± 6.6
5% glass wool	51.4 ± 3.8

## Data Availability

Data are available from the Institute of Materials Engineering, Faculty of Material Engineering and Physics, Cracow University of Technology, Jana Pawła II 37, 31-864 Cracow, Poland.
